# Association between Soluble Urokinase-Type Plasminogen Activator Receptor Levels and Chronic Kidney Disease: A Systematic Review and Meta-Analysis

**DOI:** 10.1155/2019/6927456

**Published:** 2019-11-26

**Authors:** Tiankui Shuai, Peijing Yan, Huaiyu Xiong, Qiangru Huang, Lei Zhu, Kehu Yang, Jian Liu

**Affiliations:** ^1^Department of Intensive Care Unit, The First Hospital of Lanzhou University, Lanzhou 730000, China; ^2^Evidence-Based Medicine Center, School of Basic Medical Sciences, Lanzhou University, Lanzhou 730000, China; ^3^The First Clinical Medical College of the First Hospital of Lanzhou University, Lanzhou 730000, China; ^4^Institute of Clinical Research and Evidence Based Medicine, The Gansu Provincial Hospital, Lanzhou 730000, China; ^5^Evidence Based Social Science Research Center, Lanzhou University, Lanzhou 730000, China; ^6^Institute of Evidence Based Rehabilitation Medicine of Gansu Province, Lanzhou 730000, China; ^7^Key Laboratory of Evidence Based Medicine and Knowledge Translation of Gansu Province, Lanzhou 730000, China

## Abstract

**Background:**

Chronic kidney disease (CKD) has become a global public health problem with a high prevalence and mortality. There is no sensitive and effective markers for chronic kidney disease. Previous studies proposed suPAR as an early predict biomarker for chronic kidney disease, but the results are controversial. Therefore, the purpose of the current meta-analysis is to evaluate the association between suPAR and CKD.

**Methods:**

We searched the PubMed, Embase, Cochrane Library databases, and Web of Science before May 1, 2019. The search was based on the key words including suPAR and CKD. Data are extracted independently according to standard format, and quality analysis is performed. We extracted the concentration of suPAR and hazard rate (HR) values of mortality, cardiovascular disease, and end-stage renal disease.

**Results:**

There were 14 studies fulfilling the criteria. The concentration of suPAR was higher in patients with CKD than that in the control group (*P* < 0.001; SMD: −2.17; 95% CI: −2.71, −1.63; *I*^2^ = 67.4%). SuPAR had a higher risk of mortality (*P*=0.001; HR: 1.72; 95% CI: 1.24, 2.39; *I*^2^ = 68.0%). The higher suPAR level increased the risk of cardiovascular disease (*P* < 0.001; HR: 3.06; 95% CI: 2.21, 4.22; *I*^2^ = 0.0%) and the risk of end-stage renal disease (*P* < 0.001; HR: 1.40; 95% CI: 1.22, 1.60; *I*^2^ = 0.0%).

**Conclusions:**

Monitoring suPAR concentrations may be used for early diagnosis and prognosis for patients with CKD, and the higher suPAR increased the risk of mortality, cardiovascular events, and end-stage renal disease.

## 1. Introduction

Chronic kidney disease (CKD) is a public health problem that affects about 800 million people worldwide [1]. Global burden of disease in 2016 indicated that CKD in men aged 15–49 was responsible for 1.94% of the global deaths, 0.81% of disability life-years, and 1.47% of disability adjustments [2]. In addition, its morbidity has risen annually [2]. CKD is closely related to end-stage renal disease (ESRD) and cardiovascular disease (CVD) [3, 4]. Urinary protein excretion rate and glomerular filtration rate (eGFR) are used to screen for kidney disease; however, in the early stage of kidney injury, urinary protein excretion and eGFR are not significantly reduced and, therefore, cannot detect early loss of kidney function [5, 6]. Zeier and Reiser [7] demonstrated that kidney function was partially lost before symptoms appeared, delaying the diagnosis and treatment of CKD. Thus, it is essential to choose the proper biomarkers to enable early recognition of CKD and to assess patient prognosis.

Soluble urokinase-type plasminogen activator receptor (suPAR) is the circulating form of the three-domain membrane-bound receptor. It is expressed on a variety of cells, such as podocytes, immunocompetent cells, and endothelial cells [8]. A previous study reported that suPAR might be a biomarker for kidney disease, as well as inflammatory and immune diseases [9]. Evidence also showed a wide use in sepsis, diabetes, and systemic lupus erythematosus [10, 11]. Several studies have suggested that suPAR could be a predictive factor for CKD and even for cardiovascular outcomes in patients with mild-to-moderate CKD and ESRD [12–14]. However, Stephen S. Hall et al. reported that it is still controversial whether suPAR can predict the occurrence of CKD [15]. Most of the current studies were nonrandomized control trials (RCT) with small sample size, so high-quality meta-analyses have been increasingly regarded as one of the key tools for achieving evidence [16, 17]. Moreover, this is the first meta-analysis conducted to explore the relationship between suPAR and CKD and its complications.

## 2. Methods

This meta-analysis was conducted in accordance with the Preferred Reporting Items for Systematic Reviews and Meta-Analyses (PRISMA) statement [18–20]. A Measurement Tool to Assess Systematic Reviews (AMSTAR 2) was used to assess the methodological quality [21, 22].

### 2.1. Search Strategy

Two authors (STK and YPJ) searched PubMed, Embase, Cochrane Library databases, and Web of Science databases independently from inception to May 1, 2019, with no language restriction. All relevant studies that described the relationship between suPAR and CKD were searched. The key words used to search PubMed were: “soluble urokinase plasminogen activator receptor” OR “suPAR” AND “CKD” OR “segmental glomerulosclerosis, focal” OR “glomerulosclerosis, focal” OR “focal glomerulosclerosis” OR “sclerosing glomerulonephritides, focal” OR “hyalinosis, segmental glomerular”. Conflicts were resolved by discussion between the two authors until a consensus was reached.

### 2.2. Inclusion and Exclusion Criteria

The inclusion criteria were original studies with sufficient data for extraction, suPAR levels obtained from blood samples, and patients diagnosed with CKD.

Reviews, case reports, commentaries, conference abstracts, and animal research, as well as patients included in original studies who were not diagnosed with CKD, were excluded.

ESRD was defined as the initiation of chronic dialysis or renal transplantation or irreversible development of estimated glomerular filtration rate (eGFR).

### 2.3. Data Extraction and Quality Assessment

Two authors (STK and YPJ) screened the title and abstract of the articles and read the full text of the potentially eligible studies. Relevant information, including the first author, published year, study design, location, ages, gender, diagnostic criteria, the suPAR levels, albumin, and mortality, was extracted by three authors (STK, XHY, and HQR) independently. Disagreements were dealt with by discussion between the three authors.

The quality of cohort studies was assessed by the Newcastle Ottawa Scale (NOS), and case-control studies were assessed using the Agency for Healthcare Research and Quality (AHRQ) criteria. When the opinions were not unanimous, the fourth author (ZL) discussed and resolved the differences of opinion. The results are shown in Table 1. Detailed scores are given in Table 1 of the Appendix.

### 2.4. Data Analysis

We used STATA 15.0 to analyze the data. For predicting suPAR concentration in CKD and normal controls, standardized mean differences (SMD) and 95% confidence intervals (CI) were used to predict suPAR concentrations in CKD patients and normal controls. Hazard ratios (HR) and 95% confidence intervals were used to assess combined mortality, ESRD, CVD, urinary protein, and eGFR. Heterogeneity was assessed by *I*^2^ and *P* values among the studies. *I*^2^ values from 0% to 50% indicated low heterogeneity, 51% to 75% indicated moderate heterogeneity, and more than 75% indicated high heterogeneity. If the heterogeneity was small, we used the fixed benefit model. Conversely, when the heterogeneity was large, we used the random effect model. For moderate heterogeneity and high heterogeneity, we examined heterogeneity sources through subgroup analysis. A sensitivity analysis was conducted on each of the included studies to determine the impact of individual studies on the overall experimental results. *P* values <0.05 indicated statistical differences. We used Begg's test, Egger's test, and a funnel plot to test publication bias.

## 3. Results

### 3.1. Search Results and Characteristics of the Included Studies

**Figure 1:**
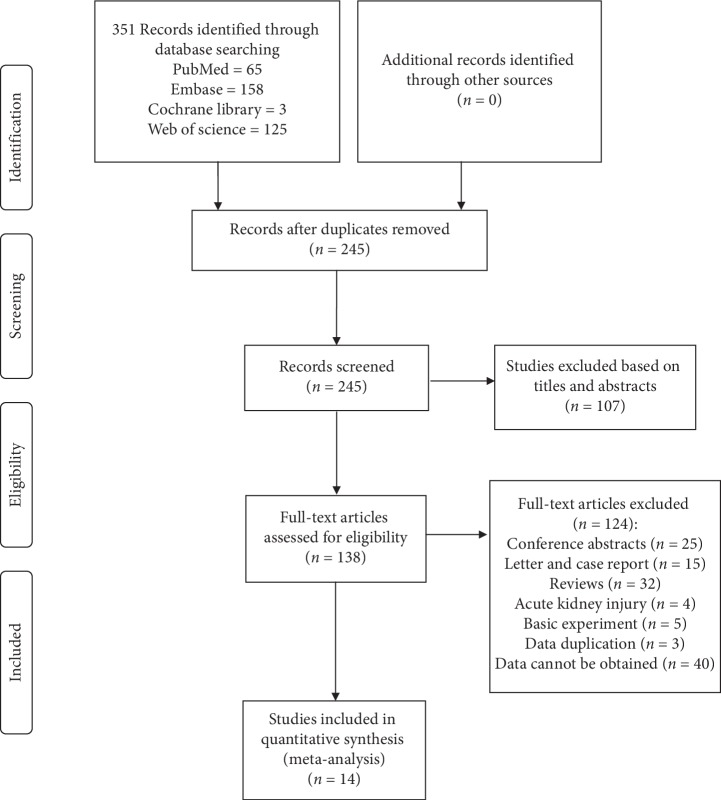
PRISMA (preferred reporting items for systematic reviews and meta-analyses) flow diagram and exclusion criteria.

From searches conducted up to May 1, 2019, a total of 351 studies, including 65 from PubMed, 158 from Embase, three from the Cochrane Library, and 125 from the Web of Science database, were identified. After reading the titles and abstracts, 107 articles were excluded. After reading the full texts, 14 articles remained [12–14, 23–33]. One hundred and twenty-four texts were excluded for incomplete data. Other studies were excluded because the contents of the research did not meet our inclusion criteria, represented the contents of a conference, or were basic research (mainly including laboratory and animal experiments). The specific inclusion and exclusion criteria are shown in Figure 1.

The 14 chosen studies included 10766 patients, comprising nine cohort studies and five cross-sectional studies. We extracted the contents of the studies, including the countries and regions of origin, patient age and gender, the quality of the studies, the classification of CKD, the etiology of CKD, and other data. The main causes of CKD were chronic glomerulonephritis, interstitial nephritis, polycystic kidney disease, secondary amyloidosis, hypertensive nephropathy, diabetes mellitus, obstructive nephropathy, ischemic nephropathy, renal tumors, nonglomerular diseases, and other renal diseases with unclear etiologies. The baseline data and demographics are shown in Table 1.

In order to reduce the impact of other factors on our results, we used a combination of multifactor HRs. However, the variables in each study were different. Most studies used age, gender, eGFR, body mass index, height, systolic blood pressure, and cardiovascular disease, while variables such as C-reactive protein, the use or nonuse of renin-angiotensin system inhibitors, the presence of diabetes, smoking history, and gene analysis were used by other studies. Detailed HR-related studies are described in Table 2.

### 3.2. Methodological Quality

All cohort studies were evaluated by NOS scores [34]. Most of the studies scored more than or equal to 7 points and only one article scored 6 points. At the same time, five cross-sectional studies were scored by AHRQ [34]. The results showed that none of the studies clearly explained the results of follow-up, and the overall quality evaluation results were between 6 and 10 points.

### 3.3. Data Synthesis

#### 3.3.1. CKD Patients Compared with the Normal Control Group

**Figure 2:**
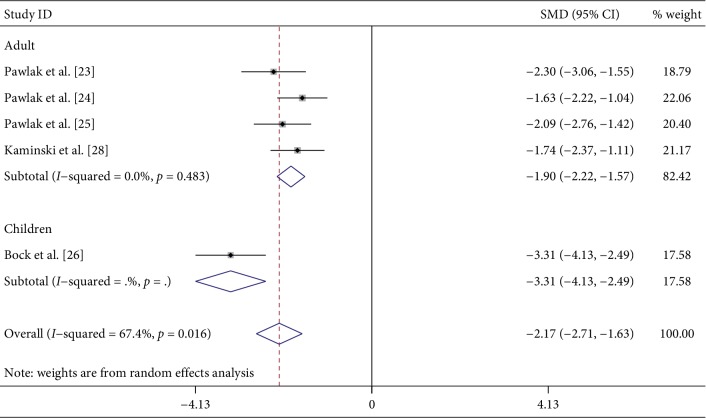
Forest plot for the concentration of suPAR between CKD and normal group.

A total of five studies [23–26, 28] compared suPAR concentrations between CKD patients and normal controls. Of the five studies, four were from Poland and the other [26] was from the United States. From an age perspective, four of the five studies were of adults and one [26] involved children. According to the staging of CKD, two studies [23, 24] involved phase 5 CKD and the other two [25, 28] included phases 1–5 CKD. The last study [26] did not clearly state the CKD phase. We used a random effect model to compare the CKD group with the normal control group and found that the CKD group had higher suPAR concentrations (*P* < 0.001; SMD: −2.17; 95% CI: −2.71, −1.63; *I*^2^ = 67.4%). Subgroup analysis of age and national region revealed no differences (Figure 2 and [Supplementary-material supplementary-material-1]). Age and national subgroup analysis showed that the CKD group still had higher suPAR concentrations (*P* < 0.001; SMD: −1.90; 95% CI: −2.22, −1.57; *I*^2^ = 0.0%). Subgroup analysis was also carried out for CKD classification. The results for CKD stage 5 were *P* < 0.001; SMD: −1.92; 95% CI: −2.58, −1.27; and *I*^2^ = 47.3% and those for CKD stages 1–5 were *P* < 0.001; SMD: −1.91; 95% CI: −2.37, −1.45; and *I*^2^ = 0.0%.

#### 3.3.2. The Effect of suPAR on Mortality

**Figure 3:**
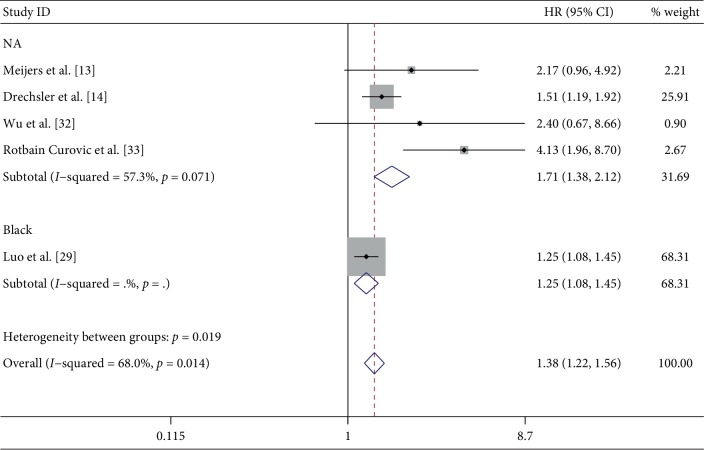
Summary Hazard Ratios (HRs) of all-cause mortality for the association between concentration of suPAR and CKD.

Five papers [13, 14, 29, 32, 33] described the multivariate HRs of suPAR levels for CKD mortality. All five studies involved adults and were from different countries and regions. The variables in these five studies were different, except for some basic variables, such as age, sex, body mass index, height, and systolic blood pressure. Most studies did not limit the ethnicity of the study population, and black Americans were clearly the subjects of one study [29]. The specific variables included in each study are shown in Table 2. By combining HRs, we determined the effect of suPAR levels on mortality (*P* = 0.001; HR: 1.72; 95% CI: 1.24, 2.39; *I*^2^ = 68.0%). In our subgroup analysis, except for the black population, high suPAR levels were associated with high mortality risk in the four other studies (*P* < 0.001; HR: 1.17; 95% CI: 1.38, 2.12; *I*^2^ = 57.3%) (Figure 3).

#### 3.3.3. The Influence of suPAR on CVD

Three articles [13, 32, 33] described the effect of suPAR levels on CVD. The study by Meijers et al. [13] reported on mild-to-moderate CKD patients and that by Wu et al. [32] reported on patients with severe CKD. The results of multivariate HR analysis showed that increased suPAR levels increased the risk of CVD (*P* < 0.001; HR: 3.06; 95% CI: 2.21, 4.22; *I*^2^ = 0.0%) ([Supplementary-material supplementary-material-1]).

#### 3.3.4. The Influence of suPAR on ESRD

The effects of suPAR levels on ESRD were studied in three articles [29, 30, 33]. A total of 4013 people were included in the three studies. The results of multivariate HR analysis showed that increases in suPAR increased the risk of ESRD (*P* < 0.001; HR: 1.40; 95% CI: 1.22, 1.60; *I*^2^ = 0.0%) ([Supplementary-material supplementary-material-1]).

#### 3.3.5. Effect of suPAR on eGFR

Two papers [27, 33] examined the effects of suPAR levels on eGFR. Schaefer et al. [27] examined the use of suPAR to predict renal function in children. Rotbain Curovic et al. [33] studied adults with type 1 diabetes. Multivariate analysis showed that high suPAR levels increased the risk of reduced eGFR (*P* < 0.001; HR: 2.51; 95% CI: 1.72, 3.66; *I*^2^ = 0.0%) ([Supplementary-material supplementary-material-1]).

#### 3.3.6. Effect of suPAR on Urinary Protein Formation

The effects of suPAR on urinary protein were studied in three articles [12, 27, 33]. The study by Schaefer et al. [27] included two cohorts that defined increased urinary protein as urinary protein excretion greater than or equal to 0.5 g/g creatinine and albumin excretion greater than or equal to 50 mg/g creatinine. Hayek et al. [12] used urinary protein test paper to define positive urinary protein as protein 1 + or higher results. Rotbain Curovic et al. [33] classified proteinuria as microalbuminuria (30–299 mg/24 hours) or massive proteinuria (more than 300 mg/24 hours). The results showed that suPAR levels affected urinary protein levels (*P*=0.007; HR: 1.83; 95% CI: 1.17, 2.84; *I*^2^ = 15.2%) ([Supplementary-material supplementary-material-1]).

#### 3.3.7. Publication Bias, Sensitivity Analysis, and Heterogeneity

We evaluated the published bias of the included studies and found no publication bias by Begg's test and Egger's test (Begg's test *P* < 0.05, Egger's test *P* < 0.05). The results of our funnel plot are shown in [Supplementary-material supplementary-material-1]. We concluded that the predictive values of suPAR levels for CKD and the effect of suPAR levels on mortality were heterogeneous, with *I*^2^ values for each of 67.4%. Considering the age of the population, the national region, the stage of CKD, and racial differences, a subgroup analysis was performed, and the results were consistent with the overall results; however, the heterogeneity declined. At the same time, in order to identify whether the results are stable, a sensitivity analysis was conducted on each individual study. The results showed that the research results were stable.

## 4. Discussion

This meta-analysis showed that suPAR concentrations were significantly different between CKD patients and normal patients, suggesting that suPAR levels could be early predictors of CKD. This study also found that higher mortality rates were associated with higher levels of suPAR in CKD patients, which may lead to a higher risk of ESRD, increased urinary protein, CVD, and the development of kidney disease.

In this study, we found higher levels of suPAR in the early stage of CKD. SuPAR is a cyclic, immune-induced signaling molecule with three domains (DI, DII, and DIII) that has become a novel research marker for multiple system damage [35]. A previous study [9] demonstrated that suPAR activated *β*3 integrin by binding to it, resulting in the disappearance of podocytes and podocyte apoptosis. Moreover, sphingomyelinase-like phosphodiesterase 3b and CD40 autoantibodies can modify and modulate the functional association of suPAR and αv*β*3 integrin [36, 37]. Several studies reported the relationship between suPAR and acute kidney injury, IgA nephropathy, and CKD [7, 38, 39]. Researchers have also found that suPAR could be a biomarker for glomerular disease [40]. Some studies [25, 26] found that patients with early CKD had higher suPAR concentrations than the normal population. Therefore, we can speculate that early detection of CKD may be possible by early monitoring of plasma suPAR concentrations. Since the original research was conducted mostly on Poles, our findings are not representative of the global population, and more research is needed in other countries or regions to verify the results of this study. The reason for the increase in suPAR is still unclear; however, relevant studies suggest that suPAR may be involved in the destruction of podocytes. Nonetheless, the specific mechanism needs further investigation.

SuPAR levels might be associated with all-cause mortality. Wlazel et al. [31] demonstrated that the predictive value of suPAR levels combined with creatinine concentrations was higher than that of suPAR alone. The presence of CVD could also be a factor in this association [41]. Furthermore, the interaction of CVD and CKD results in worse results [3]. Studies have shown that high concentrations of suPAR led to higher rates of CVD. After considering the effects of inflammatory factors and other risk factors in the patient cohorts, the authors found that suPAR was independently associated with CVD [42, 43]. They demonstrated that it could have been due to atherosclerotic inflammation and endothelial dysfunction [44, 45]. Meijers et al. [13] also reported that it might be due to malnutrition-inflammation-atherosclerosis.

At least two million patients worldwide are currently undergoing end-stage renal disease dialysis [46]. Despite advances in dialysis technology, the morbidity and mortality of dialysis patients have not improved much and remain high [32]. Our results showed that higher suPAR levels were closely related to ESRD. Lv et al. [30] conducted a stratified analysis according to the etiology of ESRD and found that suPAR concentrations were significantly elevated in the glomerulonephritis group, but that there was no significant difference in diabetic nephropathy. An inconsistent result was reported by Rotbain Curovic et al. [33]. They demonstrated that suPAR levels were significantly elevated in patients with diabetes mellitus type 1. One possible reason for the disparate results is that Lv et al. did not distinguish between type 1 and type 2 diabetes patients. Thus, further studies should clearly indicate the type of included patients to determine the relationship between suPAR and the type of diabetes.

We found that high levels of suPAR were always accompanied by low eGFRs. Early identification and management of chronic kidney disease are cost-effective and could cut the incidence of cardiovascular disease by about 50% [47]. Urinary protein and eGFR are currently the most widely used diagnostic indicators for kidney function, but they are only useful when kidney function is impaired. Several studies [28, 29] have shown that suPAR could be a possible biomarker for early diagnosis of impaired kidney function. The studies suggested that high levels of suPAR were usually attributed to an inflammatory state or reduced renal clearance [48, 49].

The heterogeneity sources of this study are as follows: first, the etiology of CKD was not uniform in the included studies. Most of the CKD cases in these studies were caused by multiple reasons. While two were attributed to diabetes, some studies did not clarify the CKD etiology. Different causes of disease, leading to different disease progression could bias the results of this study. Secondly, differences in the methods used to measure suPAR levels could also be the source of heterogeneity in this study. Such variation could occur from the use of different kits, different laboratories, and different specimen collection times. Thirdly, different baseline levels of CKD patients might also be a possible reason for variation between the included studies. Some studies included a population of CKD patients with end-stage disease, while some included not only end-stage CKD patients but also early-stage CKD patients. Age and race may also be sources of heterogeneity.

Despite some variations between the included studies, this study had some strengths. On the one hand, this is the first systematic review describing the predictive value of suPAR in CKD, CVD, and mortality. On the other hand, the adjusted HRs were used to measure the effect of suPAR. There were limitations to this study. First, patients with different CKD etiologies were included in the studies used for this meta-analysis. However, due to the limited number of original studies and data, we could not conduct subgroup analysis according to the etiological causes. Therefore, this study was unable to provide more accurate recommendations for future clinical treatment. Moreover, our meta-analysis considered the relationship between suPAR levels and urinary protein levels, but the original studies used different definitions of increased urinary protein, so the results of this meta-analysis are not equally representative of all studies. Third, because our included studies were non-RCT with small sample size, our results might be less representative. We call for further RCT studies with large sample size to test our results.

## 5. Conclusion and Future Perspectives

In conclusion, monitoring suPAR concentrations may be used for early diagnosis and prognosis for patients with CKD, and the higher suPAR increased the risk of mortality, cardiovascular events, and end-stage renal disease. Therefore, suPAR may serve as a potential biomarker for early prediction of CKD and CKD complications. Influenced by the small sample size of the original study and the type of study, our results might be less representative. Therefore, we need higher quality researches to confirm our conclusions.

## Figures and Tables

**Table 1:** The characteristic of studies (*n* = 14).

Author	Country	Study design	Year	Age (yrs)	Male	Number of population	NOS/AHRQ	Stage of CKD	Pathogeny
Pawlak et al. [23]	Poland	Cross-sectional	2007	58.5 ± 12.3	38	64	7	5	1, 2, 3, 4, 5, 11
Pawlak et al. [24]	Poland	Cross-sectional	2010	53.3 ± 15.3	43	70	6	5	1, 2, 3, 4, 5, 6, 11
Pawlak et al. [25]	Poland	Cross-sectional	2012	54.5 ± 14.3	35	60	7	1–5	1, 2, 3, 4, 6, 11
Bock et al. [26]	America	Cross-sectional	2013	12.1 ± 5.0	43	99	10	NA	1, 10, 11
Hayek et al. [12]	America	Cohort	2015	63 ± 12	2404	3683	8	NA	NA
Meijers et al. [13]	Belgium	Cohort	2015	61 ± 5.8	260	476	7	1–4	NA
Drechsler et al. [14]	Germany	Cohort	2017	66 ± 8	635	1175	6	5	6
Schaefer et al. [27]	European	Cohort	2017	11.9 ± 3.5	560	898	7	NA	1, 10, 11
Kaminski et al. [28]	Poland	Cross-sectional	2018	52.9 ± 15.7	26	65	7	1–5	NA
Luo et al. [29]	America	Cohort	2018	55 ± 11	582	955	6	NA	NA
Lv et al. [30]	China	Cohort	2018	48.2 ± 13.8	1402	2391	7	3–4	1, 6, 11
Wlazel et al. [31]	Poland	Cohort	2018	66.7 ± 13	42	64	7	4–5	2, 3, 5, 6, 7, 8
Wu et al. [32]	China	Cohort	2018	52.0 ± 14.3	53	99	7	4–5	1, 3, 5, 6, 7, 9, 11
Rotbain curovic et al. [33]	Denmark	Cohort	2019	56 ± 12	178	667	7	NA	6

1, chronic glomerulonephritis; 2, interstitial nephritis; 3, polycystic kidney disease; 4, secondary amyloidosis; 5, hypertensive nephropathy; 6, diabetes mellitus; 7, obstructive nephropathy; 8, ischemic nephropathy; 9, renal tumor; 10, nonglomerular diseases; 11, other renal disease; NA : not applicable.

**Table 2:** Multivariate factors.

Study	HR (multivariate factors)
Hayek, S. S.	Age, sex, race, BMI, proteinuria, hsCRP, renin-angiotensin system inhibitors, DM, hypertension, hyperlipidemia, coronary artery disease, smoking, myocardial infarction

Meijers, B.	Creatinine, age, gender, SBP, smoking, DM, cholesterol, calcium, phosphate, PTH, CRP, albumin

Drechsler, C.	Age, sex, BMI, hypertension, LDL, HDL, cholesterol, antiplatelet and angiotensin-converting enzyme inhibitor, heart failure, coronary artery disease, peripheral vascular disease, diuretics, vascular access, hemoglobin, albumin, phosphate, CRP, leukocyte count, asymmetric dimethyl arginine

Schaefer, F.	Age, sex, eGFR, BMI, height, SBP, diastolic, proteinuria, cholesterol, albumin, bicarbonate

Kaminski, T. W.	AA, fibrinolytic factors, renal insufficiency markers

Luo, S.	Age, sex, BP, medication, UPCR, GFR, heart disease, smoking, CRP, APOL1

Lv, L.	Age, sex. Smoking, BMI, diabetes, hypertension, CVD, triglyceride, HDL, statin, prealbumin, hsCRP, UPCR, GFR

Wlazel, R. N.	NT-proBNP, Gal-3, hsTnT, hsCRP, cystatin C, urea, creatinine, albumin, cholesterol, LDL, calcium, phosphate, PTH, hemoglobin, ferritin, TIBC

Wu, W.	Age, dialysis vintage, calcium, phosphorus, Hb, albumin, ALP, ipth, hsCRP, diabetes, hypertension, CVD, suPAR, CACS

Rotbain curovic, V.	Sex, age, DM, LDL, Hb, SBP, BMI, smoking, proteinuria, RAASi, GFR, CRP.

BMI, body mass index; CRP, C-reactive protein; hsCRP, high-sensitivity C-reactive protein; SBP, systolic blood pressure; BP, blood pressure; DM, diabetes mellitus; LDH, low-density lipoprotein; HDL, high-density lipoprotein; AA, anthranilic acid; UPCR, 24-hour urine protein-to-creatinine ratio; PTH, parathormone; TIBC, total iron binding capacity; NT-proBNP, N-terminal prohormone of brain natriuretic peptide; Gal-3, galectin-3; hsTnT, high-sensitive troponin T; suPAR, soluble urokinase plasminogen activator receptor; ALP, alkaline phosphatase; CACS, coronary artery calcification score; Hb, hemoglobin; Ipth, intact parathyroid hormone; CVD, cardiovascular disease; GFR, glomerular filtration rate; eGFR,estimated glomerular filtration rate; RAASi, renin-angiotensinaldosterone system inhibitiors.

## Supplementary Materials

Supplementary Materials Figure S1. Subgroup analysis of forest plot for the concentration of suPAR between CKD and normal group. Figure S2. Summary Hazard Ratios (HRs) of cardiovascular disease and concentration of suPAR. Figure S3. Summary Hazard Ratios (HRs) of end-stage renal disease and concentration of suPAR. Figure S4. Summary Hazard Ratios (HRs) of estimated glomerular filtration rate and concentration of suPAR. Figure S5. Summary Hazard Ratios (HRs) of urinary protein and concentration of suPAR. Figure S6. Funnel plot.
