# Supplementary material for: Association between Soluble Urokinase-Type Plasminogen Activator Receptor Levels and Chronic Kidney Disease: A Systematic Review and Meta-Analysis
Source: Biomed Res Int. 2019 Nov 26;2019:6927456. doi: 10.1155/2019/6927456 (PMC6899318; doi:10.1155/2019/6927456)
Supplement: Supplementary Materials — Figure S1. Subgroup analysis of forest plot for the concentration of suPAR between CKD and normal group. Figure S2. Summary Hazard Ratios (HRs) of cardiovascular disease and concentration of suPAR. Figure S3. Summary Hazard Ratios (HRs) of end-stage renal disease and concentration of suPAR. Figure S4. Summary Hazard Ratios (HRs) of estimated glomerular filtration rate and concentration of suPAR. Figure S5. Summary Hazard Ratios (HRs) of urinary protein and concentration of suPAR. Figure S6. Funnel plot. [file 6927456.f1.doc]

**Supplementary Figure legends**

Figure S1. Subgroup analysis of forest plot for the concentration of suPAR between CKD and normal group

Figure S2. Summary Hazard Ratios (HRs) of cardiovascular disease and concentration of suPAR.

Figure S3. Summary Hazard Ratios (HRs) of end-stage renal disease and concentration of suPAR .

Figure S4. Summary Hazard Ratios (HRs) of estimated glomerular filtration rate and concentration of suPAR .

Figure S5. Summary Hazard Ratios (HRs) of urinary protein and concentration of suPAR.

Figure S6. Funnel plot.


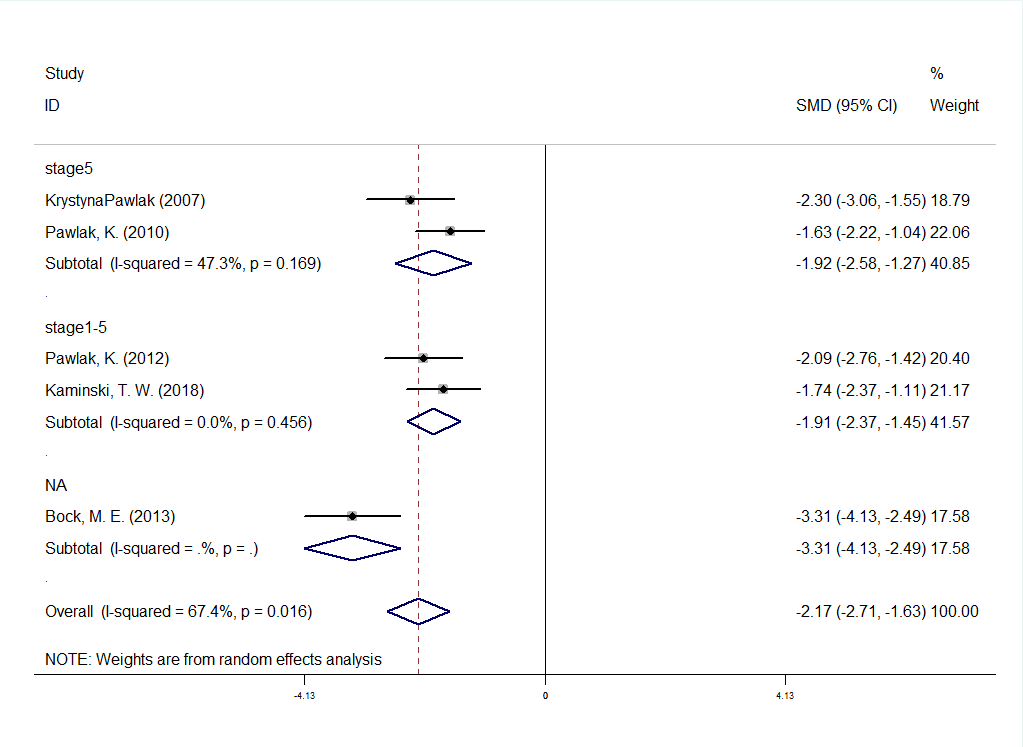


Figure S1. Subgroup analysis of forest plot for the concentration of suPAR between CKD and normal group


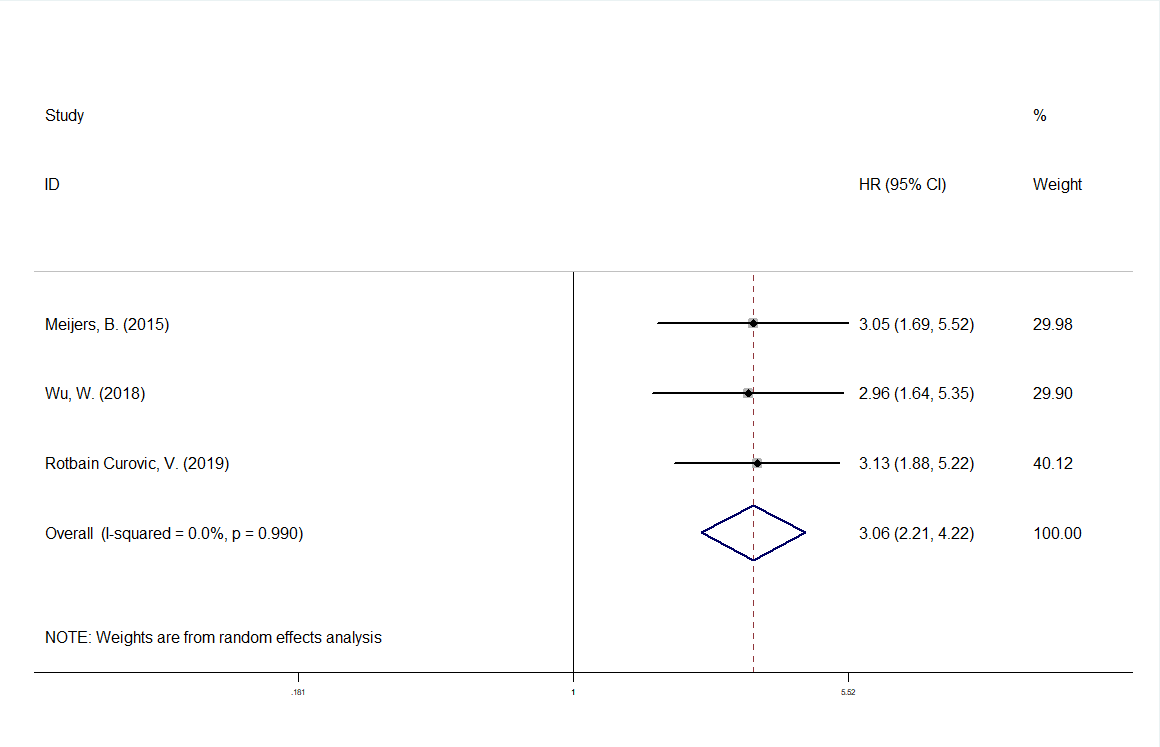


Figure S2. Summary Hazard Ratios (HRs) of cardiovascular disease and concentration of suPAR.


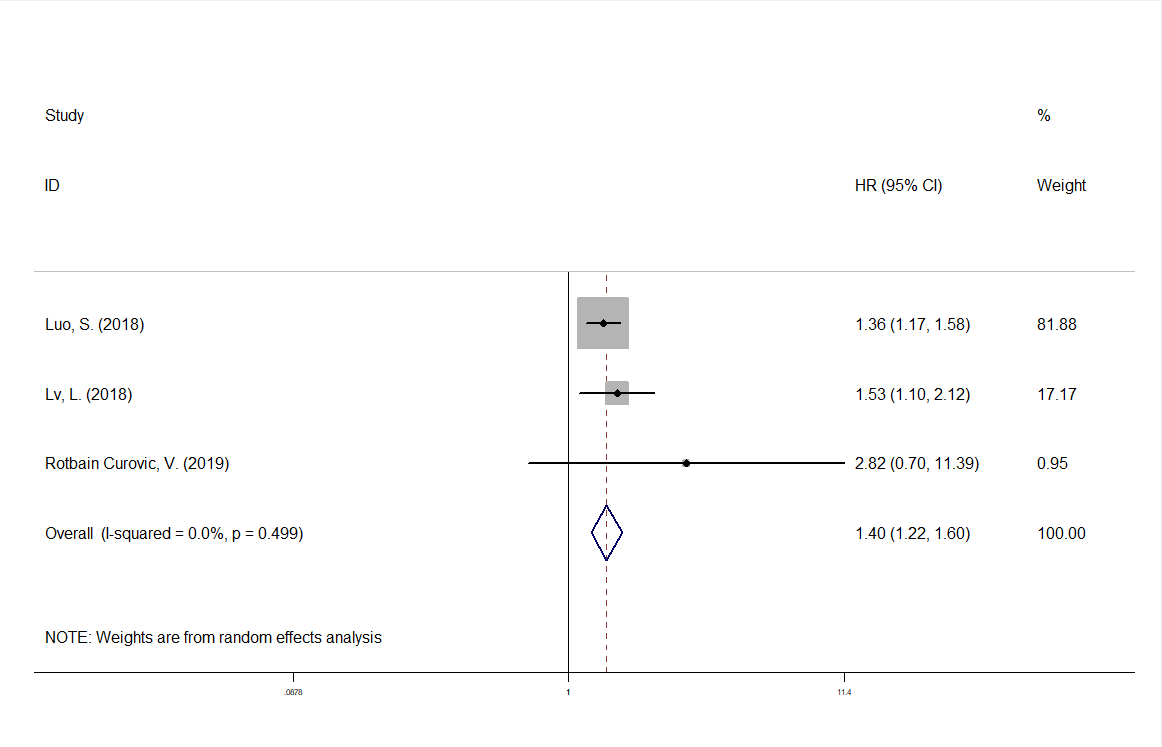


Figure S3. Summary Hazard Ratios (HRs) of end-stage renal disease and concentration of suPAR.


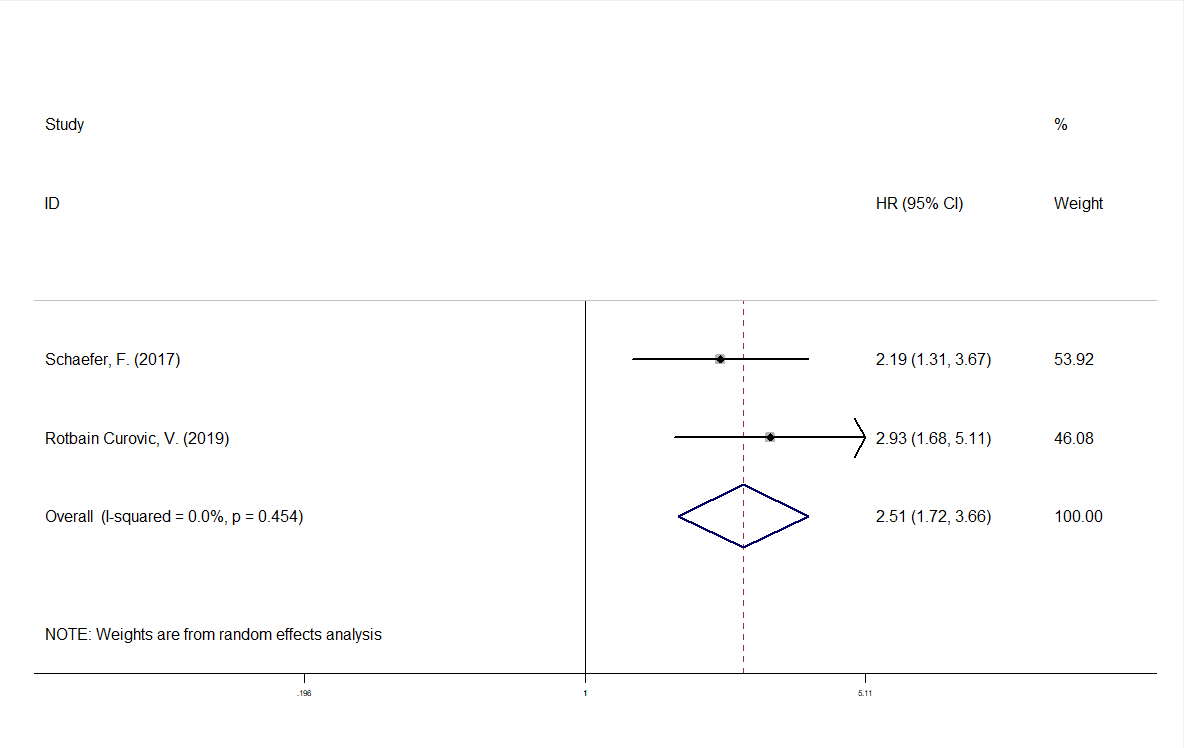


Figure S4. Summary Hazard Ratios (HRs) of estimated glomerular filtration rate and concentration of suPAR.


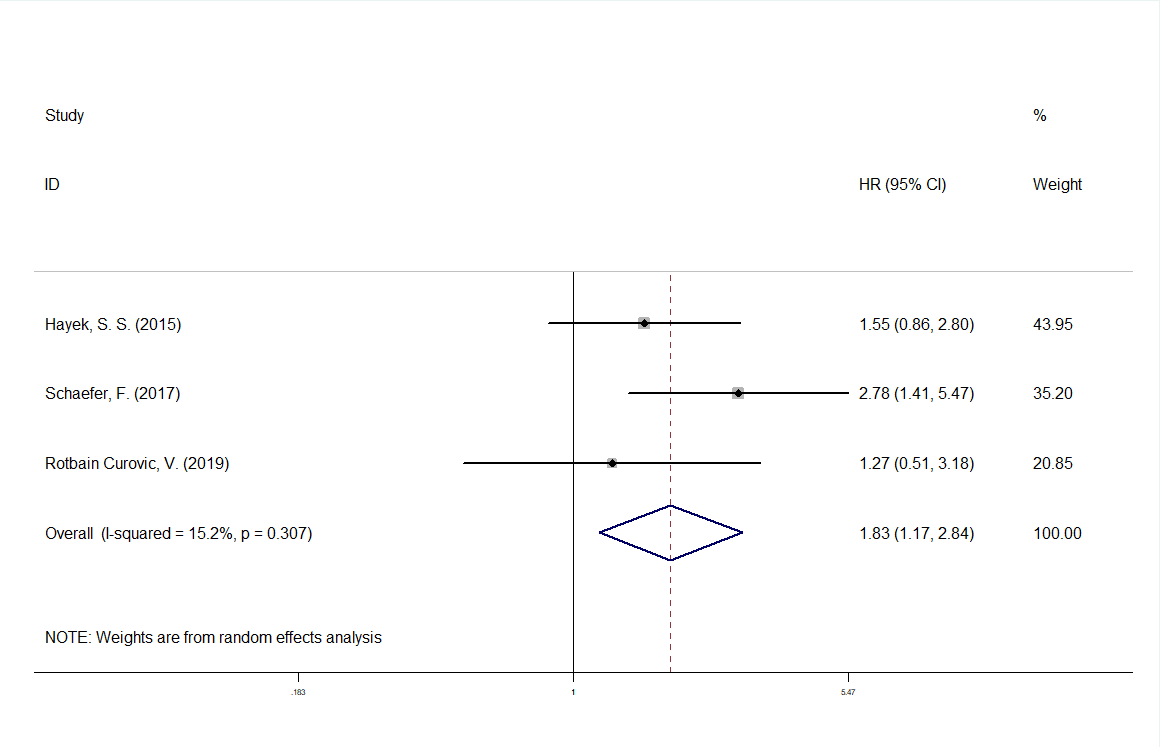


Figure S5. Summary Hazard Ratios (HRs) of urinary protein and concentration of suPAR.


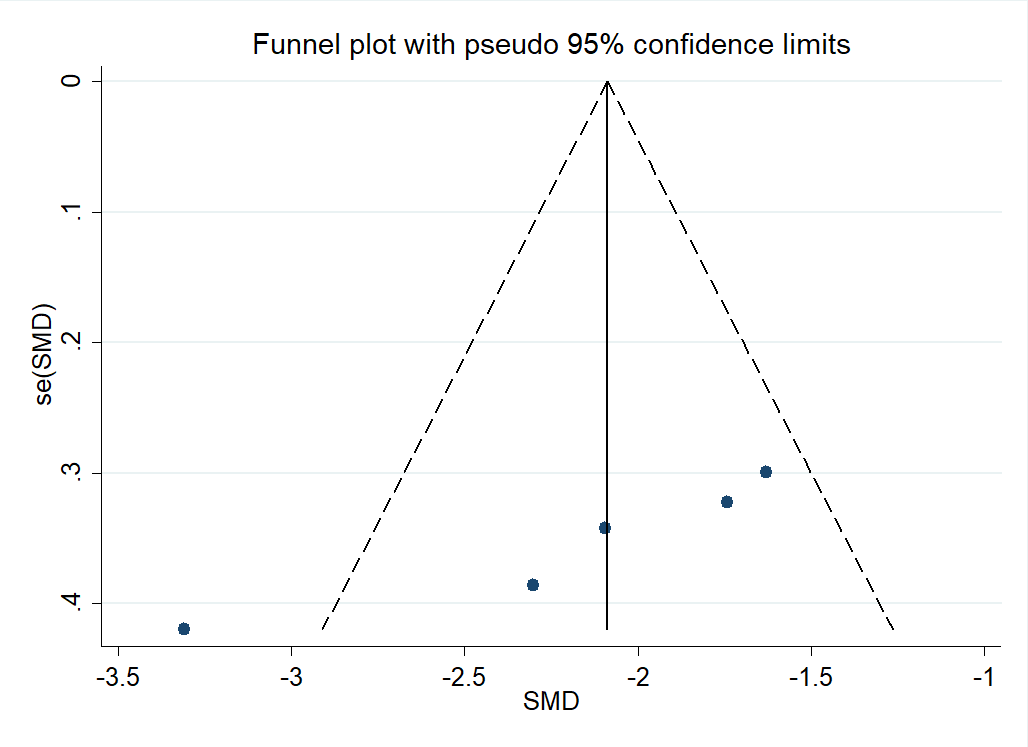


Figure S6. Funnel plot.
